# Complete Genome Sequences of Two Thermophilic Indigenous Bacteria Isolated from Wheat Straw, Thermoclostridium stercorarium subsp. Strain RKWS1 and *Thermoanaerobacter* sp. Strain RKWS2

**DOI:** 10.1128/mra.01193-22

**Published:** 2023-02-07

**Authors:** Ryan G. Bing, Daniel J. Willard, Mohamad J. H. Manesh, Tunyaboon Laemthong, James R. Crosby, Michael W. W. Adams, Robert M. Kelly

**Affiliations:** a Department of Chemical and Biomolecular Engineering, North Carolina State University, Raleigh, North Carolina, USA; b Department of Biochemistry and Molecular Biology, University of Georgia, Athens, Georgia, USA; University of Strathclyde

## Abstract

Reported here are complete genome sequences for two anaerobic, thermophilic bacteria isolated from wheat straw, i.e., the (hemi)cellulolytic Thermoclostridium stercorarium subspecies strain RKWS1 (3,029,933 bp) and the hemicellulolytic *Thermoanaerobacter* species strain RKWS2 (2,827,640 bp). Discovery of indigenous thermophiles in plant biomass suggests that high-temperature microorganisms are more ubiquitous than previously thought.

## ANNOUNCEMENT

**FIG 1:**
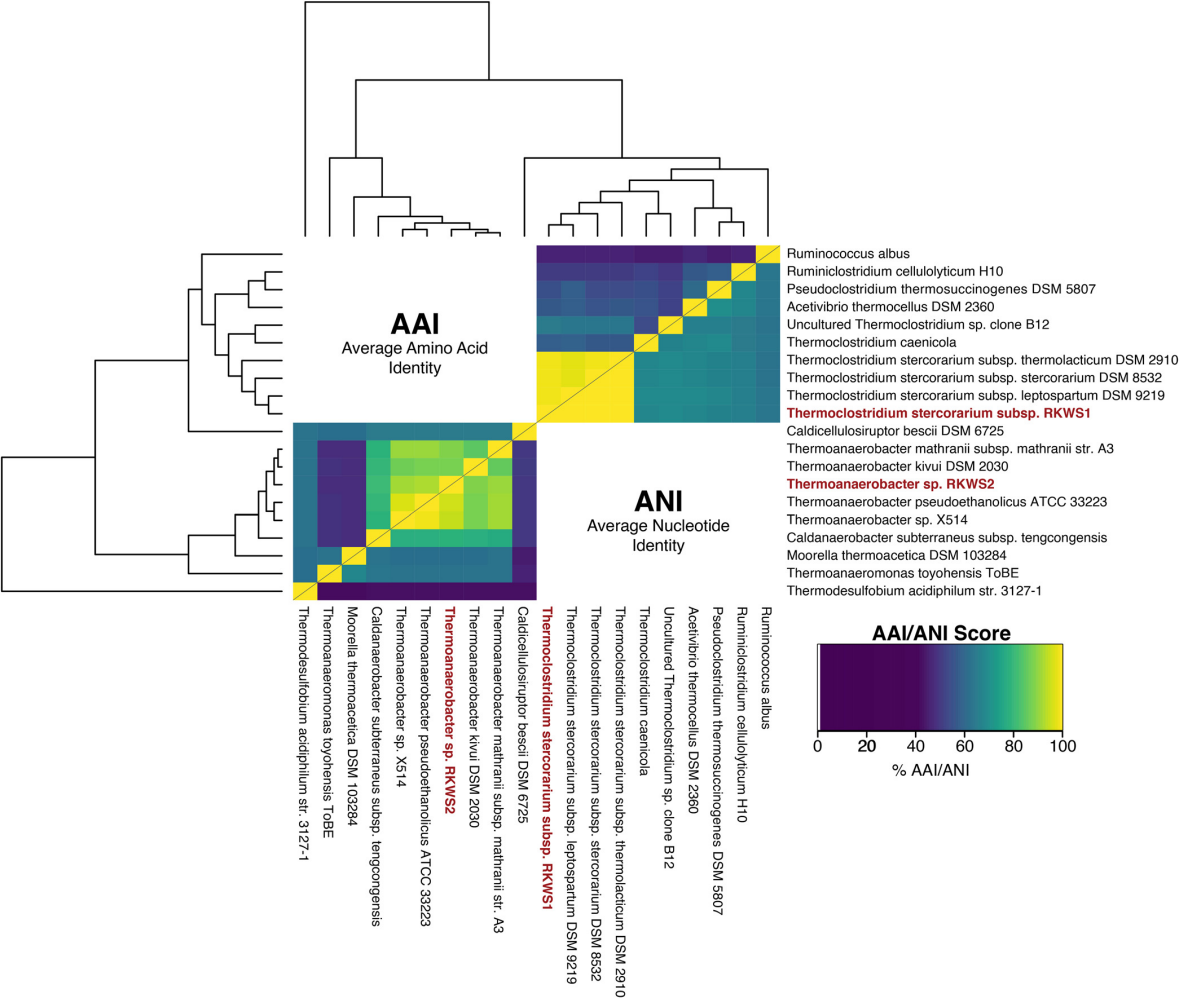
Taxonomic evaluation of wheat straw isolates. AAIs (from GET_HOMOLOGUES) (left of diagonal line) and ANIs (from OrthoANI) (right of diagonal line) for RKWS1 and RKWS2 were determined in comparison with nine other species each, spanning several taxonomic levels (RKWS1, subspecies, species, and genus; RKWS2, species, genus, and family). The taxonomic tree was generated by NCBI BLASTn TreeView with the 16S rRNA gene sequence for each species.

Microbes that degrade plant biomass have received significant attention for production of bioproducts from renewable, sustainable feedstocks; in particular, thermophilic bacteria stand out as major candidates for this process (1). Incubation of wheat straw (provided by Nature Energy, Odense, Denmark) in low-carbon (LC) medium (2) at 60°C unexpectedly revealed thermophilic microbes harbored in the plant biomass (Fig. 1) (3). Thermoclostridium stercorarium subsp. strain RKWS1 and *Thermoanaerobacter* sp. strain RKWS2 were isolated from the wheat straw culture; each consumed a variety of soluble and insoluble saccharides found in plant biomass (3).

Pure isolates were obtained via six sequential platings on solid medium for thermophilic clostridia (MTC)-cellobiose medium, starting with an indigenous mixed culture from wheat straw in LC medium (3). Colony 16S rRNA gene sequences were analyzed to track purification by PCR-Sanger sequencing or next-generation sequencing (NGS) of the 16S rRNA gene (final check) (Azenta Life Sciences). Pure isolates were stored at −80°C in 15% glycerol-MTC medium. To revive the isolates, a single 1.5-mL aliquot was thawed, inoculated into prewarmed, anaerobic MTC medium, and cultured at 60°C for 16 to 20 h in liquid MTC-cellobiose medium, with agitation at 150 rpm (New Brunswick Innova 42 shaker) (1, 3). Genomic DNA of each isolate was extracted using the Monarch genomic DNA purification kit (New England Biolabs, USA). Sequencing was performed on a MinION Mk1B system with an R9.4.1 flow cell (FLO-MIN106D) using the native barcoding kit 24 (SQK-NBD112.24) according to the manufacturer’s protocols, without DNA size selection (Oxford Nanopore Technologies, UK). DNA quantification was performed using a Qubit 4.0 fluorometer and the 1× double-stranded DNA (dsDNA) high-sensitivity (HS) assay kit (Q33231; Invitrogen). Multiplexed samples were sequenced for 72 h with live high-accuracy GPU base calling using Guppy v6.1.5 through MinKNOW v5.1.8 (Oxford Nanopore Technologies). Read quality was evaluated using MinIONQC v1.4.2 (4). Read metrics are provided in Table 1.

**TABLE 1:** Read, assembly, and quality metrics for assembled genomes from wheat straw isolates

Parameter	Data for:	
	Thermoclostridium stercorarium subsp. strain RKWS1	*Thermoanaerobacter* sp. strain RKWS2
No. of reads	2,070,295	39,215
Read *N*_50_ (bp)	3,236	8,710
Genome size (bp)	3,029,933	2,827,640
GC content (%)	42.13	34.54
Coverage of corrected reads (×)	41	39
Reads mapped (%)	99.73	99.96
Completeness (%)	95.63	96.89
Contamination (%)	2.10	1.50
No. of predicted genes	2,831	2,982
BioProject accession no.	PRJNA886731	PRJNA886731
BioSample accession no.	SAMN31140365	SAMN31140398
Genome assembly accession no.	CP110889.1	CP110888.1
SRA accession no.	SRX18233911	SRX18233910

Reads were initially trimmed as part of the Guppy base-calling workflow. High-quality reads were subsampled using NanoFilt v2.8.0, with 500-bp length and quality score Q10 read quality cutoff values (5). Corrected reads were generated from this subset using the Canu v2.2 read self-correction module (6). Flye v2.9.1 (7) assembled corrected reads into a single circularized contig for each strain. The assembled genomes were rotated to start from the *dnaA* gene predicted by Prodigal v2.6.3 (8) using the FIXSTART task in Circlator v1.5.5 (9). Read mapping was performed using GraphMap v0.5.2 (10), with sorting and indexing done by SAMtools v1.15.1 (11). Further assembly error correction and polishing were performed using Pilon v1.24 (12) and Medaka v1.0.3 (Oxford Nanopore Technologies, UK), respectively. Quality and statistics for the final assemblies were assessed using QUAST v5.2.0 (13), Pilon (–fix flag set to none) (12), and CheckM v1.2.1 using a reduced genome tree (14) (Table 1). Pilon detected no major misassembly events in the final assemblies. Finished genomes were annotated with PGAP v2022-08-11.build6275 (15). Average amino acid identities (AAIs) were calculated with GET_HOMOLOGUES v22082022 (16, 17), average nucleotide identities (ANIs) were calculated with OrthoANI v0.93.1 (18), and the taxonomic tree was generated by NCBI BLASTn TreeView (19) using 16S rRNA gene sequences. Except where specified, default parameters were used for all software.

### Data availability.

BioProject, BioSample, and genome accession numbers for RKWS1 and RKWS2 assemblies are given in Table 1. The GenBank accession numbers for other genomes used for 16S rRNA gene BLASTn analysis and AAI and ANI calculations are CP014673.1, CP014672.1, CP003992.2, NR_126170.1, CP016502.1, CP021850.1, CP001348.1, CP002403.1, ON077455.1, CP000924.1, CP009170.1, CP000923.1, CP002032.1, AE008691.1, LT838272.1, CP017237.1, CP001393.1, and CP020921.1.
